# Resolution of cor pulmonale after medical management in a patient with cblC-type methylmalonic aciduria and homocystinuria: a case report

**DOI:** 10.4076/1757-1626-2-8603

**Published:** 2009-07-30

**Authors:** Laurie Profitlich, Brian Kirmse, Melissa P Wasserstein, George Diaz, Shubhika Srivastava

**Affiliations:** 1 Department of Pediatrics, Division of Pediatric Cardiology, Mount Sinai School of Medicine1 Gustave L. Levy Place, Box 1201 New YorkNew York 10029, USA; 2 Department of Genetics and Genomic Sciences, Program for Inherited Metabolic Diseases, Mount Sinai School of Medicine1 Gustave L. Levy PlaceNew YorkNew York 10029, USA

## Abstract

We describe a 3-year-old Hispanic male with cblC-type methylmalonic aciduria and homocystinuria who presented to the emergency department with progressive tachypnea, vomiting, and edema secondary to pulmonary embolism and cor pulmonale. With aggressive medical management, there was complete resolution of right heart failure and pulmonary hypertension after 3 months. Pulmonary embolism is rare in the pediatric population. Children with cblC-type methylmalonic aciduria and homocystinuria may be at increased risk for thrombus formation and pulmonary embolism due to chronic hyperhomocystinemia, a risk factor for thrombus formation in the adult population. Aspirin therapy may be indicated in children with inborn errors of metabolism that predispose to hyperhomocystinemia.

## Introduction

**Figure 1.**
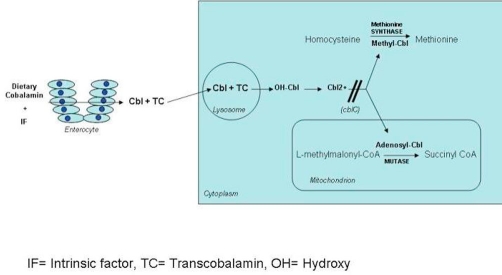
Schematic illustrating how the molecular defect in cblC results in the accumulation of methylmalonic acid and homocysteine.

CblC-type methylmalonic aciduria and homocystinuria (cblC) is an inborn error of vitamin B_12_ (cobalamin) metabolism. The disease is attributable to mutations in the gene *MMACHC* located on chromosome 1 [1]. Abnormalities in *MMACHC*’s product result in deficient intracellular conversion of cobalamin into its two functional forms: adenosylcobalamin (AdoCbl) and methylcobalamin (MeCbl) [1]. AdoCbl normally acts as a cofactor for the mutase-dependant conversion of methylmalonyl-CoA into succinyl-CoA in organic acid metabolism and MeCbl is the cofactor for methionine synthase in the re-methylation of homocysteine to methionine. Therefore, the molecular defect in cblC results in the accumulation of methylmalonic acid and homocysteine (Figure 1).

The clinical manifestations of cblC are heterogeneous, but typically include neurologic, developmental, ophthalmologic and hematologic abnormalities. Although patients with classic homocystinuria (cystathione beta-synthase deficiency) who present with isolated hyperhomocystinemia are at high risk for thromboembolic events, no thromboembolic events were reported in the largest case series of 50 patients with cblC [2,3]. There is only one case report describing an infant diagnosed post-mortem with cblC and cor pulmonale due to thromboembolism [4]. Successful medical management of cor pulmonale in this patient population has not been previously described.

## Case presentation

We describe a full-term Hispanic, male infant transferred to our neonatal intensive care unit for management of hypotonia and an abnormal urinary methylmalonic acid level. The serum homocysteine level was elevated (279 micromol/L; normal range <15 micromol/L), suggesting a disorder of cobalamin metabolism. The diagnosis of cobalamin C type methylmalonic aciduria and homocystinuria (cblC) was later confirmed through molecular testing. While in the NICU, a heart murmur was detected and an echocardiogram showed a bicommissural pulmonary valve without stenosis and normal biventricular function. He was discharged from the NICU on medical management including dietary protein restriction and hydroxycobalamin injections. He did not return for cardiac follow up during the first three years of life.

At three years of age, he presented to our emergency department with a one-week history of progressive respiratory distress, vomiting and facial and lower extremity swelling. The remainder of the medical history was noncontributory. The family reported compliance with betaine therapy, but hydroxycobalamin injections were not being given.

In the emergency department, the vital signs were pulse 130 beats/min, blood pressure 100/70 mmHg, respiratory rate 40 breaths/min and O_2_ saturation 86% while breathing room air. His O_2_ saturation improved to 99% with 5 L of oxygen by facemask. He appeared to be a small (14 kg, 25^th^ percentile), sick child in moderate respiratory distress with significant perioral cyanosis. He had periorbital and pretibial edema. His lungs had decreased air entry at the bases, but no rales were audible. Cardiac examination revealed a hyperdynamic precordium, regular rhythm, normal S1 and a loud, single S2 with no murmurs. His liver edge was palpable 8 cm below the right costal margin and the spleen tip was palpable just below the left costal margin. His distal extremities were cool with delayed capillary refill of 3-4 seconds and pulses were 1+ bilaterally.

A chest radiograph showed cardiomegaly, a right lower lobe infiltrate and a small right pleural effusion. Laboratory investigations were significant for Na 134, CO_2_ 14, BUN 42, creatinine 0.7, ALT 128, AST 247, LDH 818, d-dimer 4.34. His ABG was pH 7.33, pCO_2_ 33, HCO_3_ 17 and base deficit of −7.6. His total serum homocysteine level was 181 micromol/L (normal <15 micromol/L). The methylmalonic acid level in his urine was 90 micromol/mol Cr (normal range <2 micromol/mol Cr). CBC showed mild macrocytic anemia (hematocrit 31.2%, MCV 110.6 fL).

**Figure 2.**
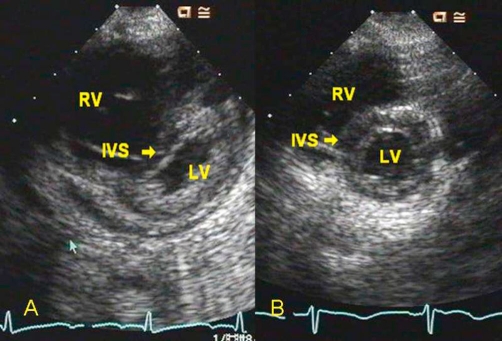
Parasternal short axis 2D echocardiographic images. **(A)** At the time of admission there was severe right ventricular (RV) dilation and evidence of RV hypertension with marked interventricular septal (IVS) flattening in systole. **(B)** At 2.5 month follow-up, the RV size is normal and there is resolution of RV hypertension with no IVS flattening in systole.

**Figure 3.**
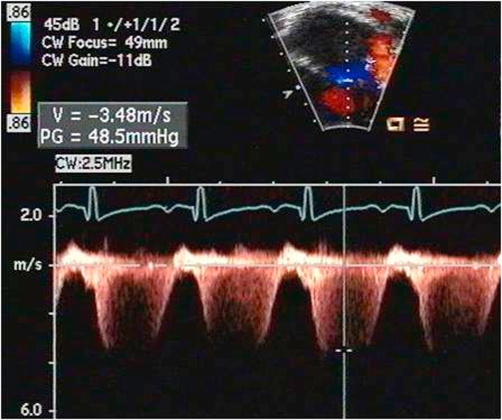
Spectral Doppler interrogation of severe tricuspid regurgitation showing a maximal instantaneous gradient of 48 mmHg. The right ventricular systolic pressure is estimated at 48 mmHg plus the right atrial pressure (more than one-half systemic pressure with systolic BP of 100 mmHg) using the modified Bernoulli equation.

**Figure 4.**
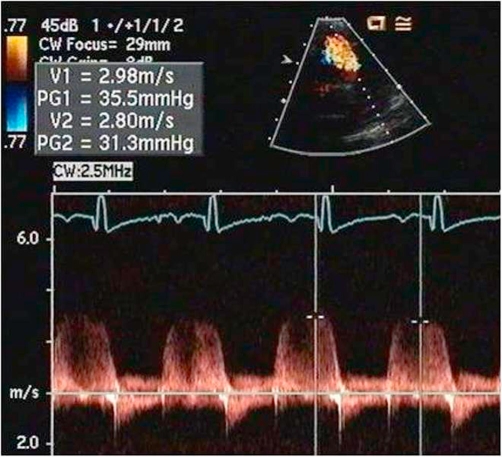
Spectral Doppler interrogation of the pulmonary regurgitant jet showing the pulmonary artery diastolic pressure to be elevated at 35 mmHg above the right ventricular end diastolic pressure.

A 12-lead EKG showed sinus tachycardia, right axis deviation and right ventricular hypertrophy. An echocardiogram showed severe pulmonary hypertension with severe right ventricular dilation (Figure 2A), severe tricuspid regurgitation, moderate pulmonary regurgitation, mild mitral regurgitation and mildly depressed left ventricular function with a shortening fraction of 24%. The right ventricular pressure was estimated to be greater than one-half of the systemic blood pressure by the tricuspid regurgitant jet (Figure 3). The pulmonary artery diastolic pressure was 35 mmHg above the right ventricular end-diastolic pressure based on the pulmonary regurgitant jet (Figure 4). Additionally, there was a small pericardial effusion.

The diagnosis of cor pulmonale secondary to pulmonary embolism was suspected. In the PICU, milrinone (0.5 mcg/kg/min), a heparin infusion (10 units/kg/hr) and furosemide (1 mg/kg IV every 8 hours) were initiated. Hydroxycobalamin (1 mg IM daily) was reinstituted and betaine therapy was optimized at a dose of 12 grams/day (850 mg/kg/day). Folic acid and pyridoxine were supplemented. A lung perfusion scan showed a large perfusion defect in the right middle lobe, but the result was indeterminate for pulmonary embolism due to poor patient cooperation. Ultrasound of the distal inferior vena cava as well as bilateral iliac, common femoral, superficial femoral and popliteal veins showed no evidence of deep vein thrombosis.

The patient tolerated the transition onto oral furosemide and digoxin. Sildenafil was initiated and his dose was increased to 5 mg (0.3 mg/kg/dose) PO every 8 hours. He was kept on low-molecular weight heparin every 12 hours for anticoagulation. Prior to discharge, an echocardiogram showed no significant improvement in his pulmonary hypertension, right heart function or degree of tricuspid and pulmonary regurgitation.

Since discharge, he has had no recurrence of cardiovascular symptoms. A repeat echocardiogram 2.5 months after discharge showed complete resolution of pulmonary hypertension with normal right ventricular size and function (Figure 2B), trivial tricuspid, mitral and pulmonary regurgitation and qualitatively normal left ventricular systolic function. His furosemide and digoxin were discontinued at that time. Sildenafil was continued for an additional 4 months and was weaned without recurrence of pulmonary hypertension. He remains on aspirin for anticoagulation and is followed regularly by our Program for Inherited Metabolic Diseases where his average total homocysteine level has been 67 micromol/L.

## Discussion

Deep vein thrombosis and pulmonary emboli are uncommon in pediatric patients [5]. A prospective Canadian registry of venous thromboembolic events (VTE) in children between the ages of 1 and 18 years showed the incidence of pulmonary embolism to be 5.3/10,000 hospital admissions or 0.07/10,000 Canadian children [6]. A prospective two-year registry of (VTE) in Dutch children less than 18 years showed the incidence of VTE to be 0.14/10,000 children [7]. Both studies found that more than 95% of children with DVT and PE had at least one associated condition predisposing them to thromboembolism [6,7]. Patients with cblC may be predisposed to thromboembolism because of the elevated homocysteine levels associated with their disease. There are no studies reporting on the relationship between hyperhomocysteinemia and thromboembolism in pediatric patients. However, mild hyperhomocystinemia is an established independent risk factor for myocardial infarction and stroke in adults, events which are often triggered by thromboembolism [8,9].

This is the first report of complete resolution of pulmonary hypertension and right heart failure after aggressive medical management in a patient with cblC. Since hyperhomocysteinemia has been shown to be an independent risk factor for thromboembolism in the adult population [10], aspirin prophylaxis may be indicated to minimize the risk of thromboembolism in pediatric patients with a predisposition for hyperhomocysteinemia. This case also underscores the importance of aggressively searching for treatable underlying conditions that predispose to thromboembolic disease, such as the inborn errors of homocysteine metabolism.

## Abbreviations

ABG: arterial blood gas
AdoCbl: adenosylcobalamin
ALT: alanine transaminase
AST: aspartate aminotransferase
BUN: blood urea nitrogen
CBC: complete blood count
cblC: Cobalamin C type methylmalonic aciduria and homocystinuria
EKG: electrocardiogram
HCO_3_: bicarbonate
IM: intramuscular
LDH: lactate dehydrogenase
MeCbl: methylcobalamin
MCV: mean corpuscular volume
NICU: neonatal intensive care unit
PE: pulmonary embolism
PICU: pediatric intensive care unit
VTE: venous thromboembolic events

## References

[bib-001] Lerner-Ellis JP, Tirone JC, Pawelek PD, Doré C, Atkinson JL, Watkins D, Morel CF, Fujiwara TM, Moras E, Hosack AR, Dunbar GV, Antonicka H, Forgetta V, Dobson CM, Leclerc D, Gravel RA, Shoubridge EA, Coulton JW, Lepage P, Rommens JM, Morgan K, Rosenblatt DS (2006). Identifcation of the gene responsible for methylmalonic aciduria and homocystinuria, *cblC* type. Nat Genet.

[bib-002] Rosenblatt DS, Aspler AL, Shevell MI, Pletcher BA, Fenton WA, Seashore MR (1997). Clinical heterogeneity and prognosis in combined methylmalonic aciduria and homocystinuria (cblC). J Inherit Metab Dis.

[bib-003] Mudd SH, Skovby F, Levy HL, Pettigrew KD, Wilcken B, Pyeritz RE, Andria G (1985). The natural history of homocystinuria due to cystathionine b-synthase deficiency. Am J Hum Genet.

[bib-004] Brandstetter Y, Weinhouse E, Splaingard ML, Tang TT (1990). Cor pulmonale as a complication of methylmalonic acidemia and homocystinuria (cbl-C type). Am J Med Genet.

[bib-005] Van Ommen CH, Peters M (2006). Acute pulmonary embolism in childhood. Thromb Res.

[bib-006] Andrew M, David M, Adams M, Ali K, Anderson R, Barnard D, Bernstein M, Brisson L, Cairney B, DeSai D (1994). Venous thromboembolic complications (VTE) in children: First analyses of the canadian registry of VTE. Blood.

[bib-007] Van Ommen CH, Heijboer H, Buller HR, Hirasing RA, Heijmans HS, Peters M (2001). Venous thromboembolism in childhood: A prospective two-year registry in the Netherlands. J Pediatr.

[bib-008] Stampfer MJ, Malinow MR, Willett WC, Newcomer LM, Upson B, Ullman D, Tischler PV, Hennekens CH (1992). A prospective study of plasma homocyst(e)ine and risk of myocardial infarction in US physicians. JAMA.

[bib-009] Bostom AG, Rosenberg IH, Silbershatz H, Jacques PF, Selhub J, D-Agistino RB, Wilson PW, Wolf PA (1999). Nonfasting plasma total homocysteine levels and stroke incidence in elderly persons: the Framingham Study. Ann Intern Med.

[bib-010] Ray JG (1998). Meta-analysis of hyperhomocysteinemia as a risk factor for venous thromboembolic disease. Arch Intern Med.

